# Food Allergen Immunotherapy in the Treatment of Patients with IgE-Mediated Food Allergy

**DOI:** 10.3390/medicina60010121

**Published:** 2024-01-09

**Authors:** Mirjana Turkalj, Adrijana Miletić Gospić, Ivona Višekruna Džidić, Ivana Banić

**Affiliations:** 1 Srebrnjak Children’s Hospital, HR-10000 Zagreb, Croatia; mturkalj@bolnica-srebrnjak.hr (M.T.); ivisekruna@bolnica-srebrnjak.hr (I.V.D.); ibanic@bolnica-srebrnjak.hr (I.B.); 2 Faculty of Medicine, J.J. Strossmayer University of Osijek, HR-31000 Osijek, Croatia; 3 School of Medicine, Catholic University of Croatia, HR-10000 Zagreb, Croatia

**Keywords:** food allergy, IgE-mediated food allergy, oral immunotherapy, OIT, sublingual immunotherapy, SLIT, epicutaneous immunotherapy, EPIT, biologics

## Abstract

The prevalence of allergic diseases, including food allergy, is increasing, especially in developed countries. Implementation of an elimination diet is not a sufficient therapeutic strategy in patients with food allergy, whose quality of life is significantly impaired. In recent years, new effective therapeutic strategies have been developed, such as the application of oral, sublingual, and epicutaneous immunotherapy. Oral immunotherapy is the most often applied strategy because of its effectiveness and ease of application, with an acceptable safety profile. The effectiveness of oral immunotherapy in patients with egg, cow’s milk, and peanut allergy has been proven both in terms of raising of the threshold and the development of tolerance, and in some patients, the development of sustainable unresponsiveness. Although oral immunotherapy is an effective treatment for food allergy, several limitations, including a long duration and a significant rate of reported adverse events, reduces its success. Therefore, new therapeutic options, such as treatment with biologicals, either as combinations with food allergen immunotherapy or as monotherapy with the aim of improving the efficacy and safety of treatment, are being investigated.

## 1. Introduction

Food allergen immunotherapy (FAIT) has been used in recent years as a novel and sustainable treatment strategy for patients with clinically relevant IgE-mediated food allergy [1,2,3]. As in the treatment of other allergic diseases, food allergen immunotherapy is the only etiological therapy. Unlike traditional approaches in which a patient with a proven food allergy is recommended keeping a strict elimination diet and using symptomatic drugs such as adrenalin in case of anaphylaxis, allergen immunotherapy is a new approach in the treatment of food allergy [3,4]. Complete elimination of the food allergen in question from the patient’s diet and avoidance of any contact with the food by ingestion, skin contact, inhalation is difficult to implement in practice due to the risk of cross-contamination or accidental exposure [2,3]. It is undoubtful that patients with food allergies have a significantly impaired quality of life, both because of the constant fear of a life-threatening reaction as well as of demanding diets that can affect their nutritional status and a balanced diet if important nutrients are avoided. Because of that, the introduction of allergen immunotherapy in patients with clinically relevant food allergies has offered a significant improvement in the management of such patients [5]. 

Certain specificities in the immune response during FAIT related to the different application routes (oral, sublingual, and epicutaneous), type of food allergen, protocols (doses and frequency of administration) appear to be associated with the effectiveness but also with the risk of side effects [5,6,7].

The primary goal of FAIT is to increase the response threshold and achieve desensitization or tolerance, and in some patients, sustained unresponsiveness (SU) even after discontinuing the regular use of allergens. This increase in the threshold is achieved after a variable period of application of increasing food allergen doses. The challenge of FAIT is to achieve SU to a food allergen, after the discontinuation of immunotherapy. Gaps persist in our understanding of the immune mechanisms of food allergy and how FAIT can lead to SU, especially since the number of patients with relapse increases over time after discontinuation of treatment [6,7]. 

The aim of FAIT is not only achieving SU or reduction in food allergen reactivity, but to attenuate life-threatening allergic reactions and reduce visits to the emergency department and hospitalization as well. Furthermore, it is not enough to achieve and prove effectiveness of FAIT, it is necessary to carry out treatment with an acceptable risk of side effects [8,9]. In selected patients in whom the protocols are adapted or in whom the allergen is applied sublingually or epicutaneously, the risk and frequency of side effects can be reduced. Unfortunately, with such routes of application, overall lower maintenance doses are reached than with oral immunotherapy. Therefore, the risk of adverse events (AE) during food immunotherapy is likely dependent on the route, maintenance doses, processing and allergenicity of the food allergen as well [7,8,9].

## 2. Mechanisms of Food Allergen Immunotherapy

FAIT is considered as an effective method for treating patients with IgE-mediated food allergies [10]. The mechanisms involved in the development of tolerance and immune modulation during FAIT are not fully understood. Development of immune tolerance and T and B cell responses changes were found in patients during FAIT [11,12]. A switch in immune responses from Th2 to Th1 cell polarization with an increase in INF-γ and a decrease in Th2 related cytokines (IL-4, IL-13) were reported. During immunotherapy exposure to continuous high doses of food allergens leads to Th2 and Th2A anergy and/or deletion and an increase in T regulatory cells which leads to the suppression of downstream allergic responses.

FAIT incompletely targets subsets of Type 2 immune cells which is a transient and non-permanent shifting [13]. Additionally, studies show that the function and number of Treg cells increase during FAIT [11,14]. In the development of peripheral tolerance Treg cells have a critical role, including the activation of specific cell subpopulations such as: inducible T regulatory (iTreg) cells, natural T regulatory (nTreg) cells and Tr1 cells and TGF-β producing Th3 cells [15]. However, the impact of FAIT on the differentiation Treg subpopulations is not well-understood. Certain studies showed an association between increased Tregs and improved outcomes in immunotherapy [16,17] while others did not prove the same effect [13,18]. A significant suppression in T follicular helper cells (Tfh) cells and transformation of follicular regulatory T (Tfr) cells following FAIT were found. Tfr cells may have important roles during FAIT and the development of immune tolerance which results in a significant decrease in Th2 responses. Modification of the Th2-mediated immune response appears to be essential for the achievement of tolerance to a particular food allergen, i.e., in the prediction of the efficacy of FAIT [19]. In FAIT with egg, high baseline levels of egg specific CD4+ Th2 cells strongly predicted failure of FAIT treatment [20]. In a study focusing on FAIT to peanut the failure to suppress Th2 response was associated with treatment failure [13]. 

Generally, every form of FAIT, i.e., oral, sublingual, and epicutaneous immunotherapy is characterized by decrease in basophil and mast cell activation [21,22]. Peanut oral immunotherapy (OIT) and milk sublingual immunotherapy (SLIT) have shown early but transient decreases in basophil activation with the loss of tolerance after the discontinuation of the immunotherapy [23,24]. During the up-dosing phase of FAIT, there is an initial rise of allergen-specific IgE levels, which is a consequence of the proliferation of allergen-specific memory B cells, followed by a gradual decrease in allergen sIgE at the end of the therapy [25]. 

Changes in the humoral response during FAIT are manifested by an increase in food protein specific immunoglobulin G, subclass 4 (IgG4) and specific immunoglobulin A (IgA) as well [26,27]. The increase in food-specific IgA may play a role in the induction of tolerance [28]. Increased levels of specific IgG4 [29] are supposed to facilitate desensitization through its binding to the inhibitory IgG receptor (FcγRIIb), thus suppressing IgE signalling pathways [29,30]. The induction of allergen specific IgG4 during FAIT seems to be a result of the IL-10 producing subpopulations of regulatory T or B lymphocytes in desensitized patients [11,31].

Decreased activation of basophils and mast cell are observed during the desensitization phase of FAIT measured by the suppression of the allergen-specific skin prick test and basophil activation test [32].

Modulation in the response of CD8+ T lymphocytes can be a predictor of response to OIT as well. The POISED study with peanut OIT showed that baseline levels of naïve CD8+ T cells and peanut -and Ara h 2-specific IgE were in positive correlation with treatment efficacy [33]. The role of dendritic cells during FAIT is not well-established. An association between positive response to therapy and decreased TNF-α producing myeloid dendritic cells (mDCs) was also reported [34]. The mechanisms related to the modulation of the immune response and the development of tolerance during FAIT are described independently of the method of exposure to the allergen. There are certain differences in the initial immune response depending on whether the allergen is introduced under the skin, orally, sublingually, or epicutaneously. The basic knowledge about the mechanisms of SLIT was published on research related to investigations of grass pollen immunotherapy in patients with allergic rhinitis [35].

Allergens delivered by SLIT are downloaded by oral Langerhans cells or myeloid dendritic cells in the oral mucosa which migrate to oral-draining lymph nodes where they promote FOXP3-positive T regulatory cells and the production of tolerogenic cytokines including IL-10 and TGF-β, and downregulate Th2 related cytokines (IL-4, IL-13) [36].

During epicutaneous application, allergens pass through epidermis and are taken up by epidermal Langerhans cells or allergens can be captured directly by dermal dendritic cells. Epidermal Langerhans cells or dermal dendritic cells migrate to skin-draining lymph nodes where they can activate T cells and other cells of adaptive immunity to induce Th2/Th1 switch and development of immune tolerance [37].

Although numerous studies have been published in recent years on the mechanisms action in FAIT, it has not yet been clarified which key changes in the immune response are related to the effectiveness of the treatment. Additional research is needed to pinpoint potential predictive biomarkers of treatment success and failure [8].

## 3. Oral Immunotherapy (OIT)

OIT represents the most common route of administration of FAIT where the allergen applied orally is immediately swallowed. It consists of daily ingestion of the food allergen dose starting below a patient’s threshold and increasing the dose over time to increase tolerance to that food [38]. The first report of OIT dates back in 1908 when Schofield successfully desensitized a 13-year-old boy with a history of anaphylactic reactions to egg by consuming gradually increasing amounts of egg, while otherwise avoiding egg completely. Although this early success has been promising, OIT was disregarded for the most part of the 20th century. However, given the several fold increase in the prevalence of food allergy in the last 30 years, OIT has very much come to focus again [39].

### 3.1. Efficacy of OIT

OIT is currently the most studied type of FAIT but due to the heterogeneity of studies (trial design, sample size, participant’s age and their baseline allergy status, dose-increase schedule, maintenance dose and therapy duration) it is difficult to compare them. In this review we try to include randomized controlled trials (RCT) on most common OIT-treated type of allergy such as milk, egg, and peanut.

#### 3.1.1. Milk OIT

The first milk OIT double-blind RCT was carried out in 2008 by Skripak et al. involving 20 children with cow’s milk allergy (CMA) [40]. All children were desensitized with median increasing reactivity threshold from baseline 40 mg to cumulative dose of 5140 mg milk protein on the end of OIT, with no change in threshold in the placebo group. AE were frequent in the OIT group, but nearly 90% were mild to moderate with no treatment requirement. OIT followed by measured dairy intake at home on daily basis led to a continuous threshold improvement, however accompanied by AE, sometimes to the previously tolerated dose [41]. In a more recent ORIMA study carried out in children with severe CMA, 50% OIT participants were desensitized with significant increase in milk threshold, but the incidence of AE, including those requiring adrenaline administration, were high [42]. In general, milk OIT carries a high risk of AE development, so efforts were made to find strategies to increase protocol safety through different, less allergenic forms of milk.

There is emerging evidence that heat-processing and food matrix could change the allergenicity of milk protein. Baking milk within a wheat matrix reduces the potential of milk protein to cause allergic reactions. Heating processes induce conformational changes in certain cow’s milk epitopes, especially whey proteins such as β-lactoglobulin, whose allergenicity significantly decreases above 90 °C, while caseins are stable to heat-treatment [43]. Nagakura et al. compared the safety and efficacy of low-dose OIT with heated milk (HM) or unheated milk (UM) in children with anaphylaxis [44]. HM includes milk powder prepared by heating cow’s milk at 125 °C for 30 s and spray-drying for 3 s, while UM refers to unheated cow’s milk sterilized at 125 °C for 2 s (UHT milk). Although the treatment efficacy was a bit lower in the HM group, the frequencies of total AE, moderate as well as severe, were significantly lower in the HM than in the UM group. Interestingly, whereas casein-specific IgG4 levels significantly increased from baseline in both groups, β-lactoglobulin-sIgG4 levels significantly increased only in the UM group. It is assumed that β-lactoglobulin-sIgG4 may require exposure to unheated β-lactoglobulin and this may be related to differences in treatment efficacy among HM and UM group.

Although OIT with baked milk is promising, the amount of proteins in such products is not standardized which has created the need for a safe product with a standard amount of protein. The iAGE-product is well-defined standardized heated and glycated milk protein product whose tolerance has been investigated in a small pilot study and showed that this product could be safe for ordinary OIT treatment in a infants and young children suffering from CMA, but future studies should confirm these results and assess the effectiveness of tolerance induction acceleration in larger samples [45].

Allergenicity of cow’s milk protein could be decreased or lost by breaking down cow’s milk proteins into short peptides by enzymatic hydrolysis, and the effect of allergenicity depend on the final peptide fragment size [43]. Partially hydrolysed formulas (pHF) consist of peptides with molecular weights of approximately < 5000 Da while extensively hydrolysed formula (eHF) contains only peptides with molecular weights < 3000 Da. In contrast to eHF, pHF is not intended for use with infants with CMA but possesses low allergenicity. OIT involving the ingestion of pHF improved tolerance to cow’s milk in children suffering from severe CMA, compared with those consuming eHF, in a safe manner [46]. Milk OIT studies discussed in text are listed in Table 1.

#### 3.1.2. Egg OIT

The first egg OIT double-blind RCT dates from 2012. Burks et al. [47] reported that 78% children in the OIT group were desensitized after 22 months, while 28% children in same group achieved SU after 24 months. At the same time, no child in the placebo group has passed the (oral food challenge) OFC test. AE occurred most frequently in the OIT group, but less than 1% were considered moderate. However, some allergic reactions were of sufficient clinical significance that approximately 15% of the children from the OIT group did not finish the treatment, mostly due to allergic reactions [47]. In the long-term OIT follow-up study the same participants were followed up for 4 years. Half of OIT-treated subjects achieved SU by year 4 with mild symptoms reported throughout the study which demonstrated that the probability of achieving SU after OIT increases with longer duration of therapy [7]. Furthermore, possibility to unlimited egg consumption lasted up to 5 years after completion of therapy in most egg-allergic children [48]. In more recent OIT studies on children with severe egg allergy, despite frequent AE, most participants in the OIT group were desensitized or partially desensitized compared with the avoidance group, which enables them to incorporate egg products into their daily diet or has improved their quality of life [49,50]. It seems that polysensitization to all 4 egg allergen molecules Gal d 1–4 is associated with poor de-sensitization responses [49].

Like with milk, allergenicity of egg proteins could be changed during processing due to protein unfolding which leads to conformational changes that can hide or destruct specific IgE binding epitopes. Generally, heating decreases the allergenicity of egg proteins, especially within a wheat matrix like cakes or biscuits [51]. The majority of egg allergic patients are tolerant to a certain amount of baked egg (BE) products, but it is questionable whether ingestion of low levels of BE boosts tolerance development, or this allergy phenotype is simply predictive of tolerance development [52,53]. It appears that in children allergic to unbaked egg but tolerant to BE, egg OIT was preferable to BE ingestion for inducing SU [6]. Egg OIT may also be safer and more effective in BE tolerant than in BE reactive children [6]. Sensitization to heat resistant egg white allergen Gal d 1 (ovomucoid) may be a useful predictor of clinically reactive BE allergy phenotype [54]. Egg OIT studies presented in text are listed in Table 2.

#### 3.1.3. Peanut OIT

The prevalence rate of peanut allergy has increased several-fold during the past decades, especially in Westernized nations where it currently affects 1–4.5% children. It is one of the most frequent triggers for fatal anaphylaxis and generally persists to adulthood, which is why response management is crucial [55]. For this reason, most OIT studies have been carried out with the peanut allergen. The first peanut OIT double-blind RCT was conducted by Varshney in 2011 on a small sample of 25 children younger than 16 years of age with peanut allergy. All OIT-treated subjects, but no one in placebo group, were desensitized to the cumulative dose of 5 g peanut protein (equivalent of approximately 20 peanuts). This regimen was well-tolerated, accompanied with only mild clinically relevant symptoms during the build-up phase, but the trial did not include patients with a history of severe anaphylaxis [56]. The STOP II study reported that OIT to peanut was successful in the induction of desensitization in most children suffering from peanut allergy of any severity, raising the reactive threshold at least 25 times so that nearly 90% of participants can tolerate daily ingestion of 800 mg of protein (5 peanuts) which significantly improved their quality of life. Side-effects were mild in most participants [57]. The POISED study has shown that OIT induces desensitization to 4 g of peanut protein in most peanut-allergic individuals, but discontinuation, or even reduction to 300 mg per day, decreases the probability of tolerating peanut at previously achieved thresholds indicating the importance of continuing daily allergen ingesting.

Biomarkers associated with SU were higher baseline peanut specific IgG4/IgE ratio and lower Ara h 2 IgE and basophil activation responses [33].

Most OIT trials included school age children, but the hypothesis that early immunotherapy interventions potentially disrupt peanut allergy due to the plasticity of a relatively immature immune response, encouraged Vickery et al. to treat peanut allergic children under 3 years of age. They reported that 78% of subjects receiving OIT demonstrated SU to peanut. Interestingly, low-target maintenance dose of peanut protein (300 mg per day) was as effective as high-target maintenance dose (3000 mg per day) suggesting that low-dose therapy achieved immunoregulation and a high rate of SU in young children [58]. They also reported that SU was clearly associated with low baseline peanut sIgE levels. Children who completed OIT were approximately 19-fold more likely to begin eating peanut-containing foods than matching peanut-allergic controls practicing avoidance. The IMPACT trial confirmed that starting the peanut OIT in children younger than 4 years was associated with an increase in both desensitization and SU. The majority (71%) OIT-treated children were desensitized to 5 g of peanut protein, while fewer patients (21%) achieved SU. Nevertheless, in OIT-treated participants, there was substantial increase in peanut tolerability compared with baseline 25 mg peanut protein at study entry and this was not seen in the placebo group. An inverse relationship between the age at screening and SU was also observed in OIT participants with the best outcome noted in the youngest children under 2 years (71% SU). The trial concluded that SU was predicted by younger age and lower baseline peanut- specific IgE [59]. In both trials most participants had at least one OIT dosing reaction, mostly mild to moderate. 

Apart from the conventional food form usually used for OIT, commercial standardized products could also be used for this purpose. The ARC001 study has been the first phase 2 double-blind placebo RCT peanut OIT trial assessed the efficacy and safety of AR101, oral biologic drug product with defined peanut protein profile, intended to reduce clinical reactivity to peanut in children and young adults with peanut allergy. AR101 has met its primary endpoint, demonstrating desensitization to 300 mg peanut protein in 79% of the AR101 group compared with 19% in placebo group, which is more than the amount of peanut typically triggering a reaction with accidental ingestion (approximately one and a half peanuts). AE occurred in nearly all AR101 subjects, but more than 95% were mild [60]. In a phase 3 double-blind placebo-RCT called PALISADE which has been carried out on children and adolescents who were highly allergic to peanut, AR101 demonstrated higher efficacy compared with placebo in children and adolescents aged 4 to 17 years, but this effect in adult participants was not significant [61]. An open-label follow-up study to PALISADE, called ARC004, explored long-term treatment beyond 1 year using 300 mg of Peanut [*Arachis Hypogaea*] allergen powder-dnfp (PTAH), formerly AR101. The ARC004 trial reported that daily dosing cohorts appeared to have higher desensitization and lower AE rates than non-daily dosing cohorts, indicating that, in children and adolescents, continued daily treatment with PTAH beyond 1 year is associated with continued and improved efficacy and safety [62]. Peanut-allergic participants evaluated after ~1.5 and ~2 years of daily PTAH demonstrated an increased tolerance to peanut protein with a potential for lower frequency of AEs, which, over time, positively affected the quality of life in c children and adolescents with peanut allergy, as well as their caregivers [63]. Studies included OIT with peanut discussed in text are listed in Table 3.

#### 3.1.4. OIT to Other Food Allergens

OIT studies with other food allergens are scarce, especially those of RCT design. In one of the rare double-blind RCT with wheat, high-dose wheat OIT induced desensitization in most participants after 1 year and SU in 13% of subjects after 2 years. Compared with egg OIT, efficacy of wheat OIT was lower, but the safety was similar [64].

In general, OIT is carried out to one food allergen at a time, but a multifood OIT study reported that desensitization to 1 food or multiple foods simultaneously through OIT appears to be safe and feasible using the OIT protocol that has been established [65].

### 3.2. Safety of OIT

OIT studies for food allergy are promising, but treatment is frequently complicated by AE including severe reaction requiring epinephrin. Although AEs are mainly related to the build-up phase, they also appear in the maintenance phase, sometimes to a previously tolerating dose, usually accompanied by certain risk cofactors like exercise, viral infection, or menses. This often causes anxiety and fear and is one of the main reasons for withdrawing from the study. Therefore, interventions to improve OIT for patients are needed. Changing patient mindsets about treatments and symptoms, encouraging the mindset that symptoms can signal desensitization, is a potential route to help patients cope with challenging medical treatments and may benefit both patient experience and physiological treatment outcomes [66]. Even when successful, OIT has limited long-term efficacy as benefits usually decrease when treatment is discontinued. 

## 4. Sublingual Immunotherapy (SLIT)

Sublingual immunotherapy (SLIT) for food allergy involves placement of allergen solution (µg to mg) under the tongue on daily basis. The main aim is to achieve allergen-specific desensitization [38]. SLIT can represent a promising method in clinical use because of its simple administration, very low doses of allergen that are used and its overall safety profile. Its efficacy and safety profile are reviewed in this paper (Table 4).

### 4.1. Efficacy of SLIT

SLIT, as a method of immunotherapy has been studied in the treatment of kiwi, apple, peach, hazelnut, peanut, and milk allergies. SLIT for food allergy treatment was first described in 2003 [67] where a subject underwent SLIT with kiwi extract and was successfully desensitized. Birch pollen–related apple allergy (BPRFA) represents one of the most prevalent food allergies in adult patients. Symptoms appear due to birch pollen allergen Bet v 1 which is highly cross-reactive with Mal d 1 (apple protein). Study of Kinaciyan et al. [68] demonstrated that equal doses of Bet v 1 and Mal d 1 allergens induce different clinical and immunologic outcomes. SLIT with specific Bet v 1 did not result with significant desensitization outcomes in patients with Mal d 1 allergy. This study showed efficacy in treatment of BPRFA with SLIT using the Mal d 1 (apple allergen) but not using the Bet v 1 (birch allergen). Double-blind, placebo RCT for hazelnut from 2005. and peach allergy from 2014. found a significant increases in tolerance to hazelnut and peach extract after sublingual immunotherapy [69,70]. 

**Table 4:** Review of efficacy and safety in SLIT.

Author (Year)	Type of Study	Allergen	Participants	Duration	Efficacy	Safety
**Kinaciyan et al. (2018) [68]**	Double-blind, placebo-RCT	Apple protein	n = 60 (aged 18–65 y) 1:1:1 = placebo:rMal d 1:rBet v 1	16 weeks	rMal d 1 vs placebo and rBet v 1 (*p* = 0.001 and *p* = 0.038) SLIT rMal d 1 enhanced IgG4/IgE ratios (*p* = 0.012).	Mainly local AE to both formulations
**Enrique et al. (2005) [69]**	Double-blind placebo-RCT	standardized hazelnut extract	n = 23 (19–53 y) Active group n = 12 Placebo group n = 11	12 weeks	Median SCD hazelnut SLIT *p* = 0.02 50% active group reached highest dose (20 g)	AE Mild; Systemic reactions 0.2%
**Garrido-Fernández et al. (2014) [70]**	Double-blind placebo-RCT	peach extract	n = 31 (18–65 y) Treatment group:placebo = 2:1	6 months	Median SCD *p* = 0.002 3-fold improvement in tolerance in active group *p* = 0.065	No data. All subjects that started treatment also have finished it
**Keet et al. (2012) [71]**	Open- label exploratory RCT	milk	n = 30 (6–17 y)	60 weeks	1/10 SLIT/SLIT group, 6/10 SLIT/OITB group, 8/10 SLIT/OITA group—SCD DBPCFC 8-g (*p* = 0.002, SLIT vs. OIT) End of study: *p* = 0.09 SLIT vs. OIT	Symptoms 29% of SLIT doses and 23% of OIT doses. no significant differences in the rate of total AE SLIT and OIT *p* = 0.73, 0.70, and 0.50, respectively Multisystem symptoms OIT vs. SLIT *p* < 0.001
**Kim et al. (2011) [72]**	Double-blind placebo-RCT	peanut	n = 18 (1–11 y) Peanut SLIT n = 11 Placebo n = 7	12 months (6 months build-up and 6 months MD)	Median SCD peanut SLIT vs. placebo *p* = 0.011	Transient oropharyngeal itching most common AE 0.26% antihistamine treatment 0.02% doses required albuterol for mild wheezing
**Kim et al. (2019) [73]**	Open-label extension RCT	peanut	n = 48 (1–11 y) 2-mg peanut SLIT MD	5 years	67% SCD ≥ 750 mg on DBPCFCs. median SCD 1750 mg 25% (12/48) 5000-mg DBPCFC; 10/12 SU after 2 to 4 weeks	AE 4.78%; transient oropharyngeal itching most common Antihistamine use 0.21% No epinephrine
**Kim et al. (2023) [74]**	Open-label, prospective RCT	peanut	n = 54 (1–11 y) 4 mg peanut SLIT MD	48 weeks	Mean SCD (0–48 month) *p* < 0.0001 36% SCD of 5000 mg 70.2% SCD ≥ 800 mg	Dosing AE 4% of doses
**Fleischer et al. (2013) [75]**	Multicenter placebo-RCT	peanut	n = 40 (12–40, median age 15 y) Placebo group n = 20 Intervention group n = 20	The first phase 44 weeks (68 weeks data)	Week 44: RR intervention group vs placebo *p* < 0.001 median SCD week 44 vs. baseline in intervention group *p* < 0.01 Week 68: median SCD week 68 vs. 48 *p* = 0.05, week 68 vs. baseline *p* = 0.009 Week 44 Crossover: median SCD *p* = 0.02	Week 44: transient oropharyngeal itching most common AE 1.1% of total doses required treatment. Crossover High Dose subjects: 2.9% doses required treatment 1 subject had anaphylaxis
**Burks et al. (2015) [76]**	Long-term follow-up RCT	peanut	n = 40 (12–40 y)	From week 68 to 164	4/37 (10.8%) of SLIT participants fully desensitized to 10 g of peanut powder and SU	98% of the doses were tolerated without AE no severe symptoms no epinephrine

RCT—randomized controlled trial; SLIT—sublingual immunotherapy; y—years; MD—maintenance dose; SCD—successfully consumed dose; RR—response rates; AE—adverse events; SU—sustain unresponsiveness; DBPCFC—double-blind placebo-controlled food challenge; OIT—oral immunotherapy.

In RCT of Keet et al. [71] children with CMA underwent SLIT or SLIT escalation followed by OIT. After initial DBPCFC and SLIT escalation, participants either continued SLIT to 7 mg daily or began OIT (1000 mg allergen OITB group or 2000 mg the OITA group) using milk protein. After maintenance period at week 12 and week 60 participants underwent DBPCFC with 8 g of milk protein. A total of 1/10 participants in the SLIT group, 6/10 participants in the SLIT/OITB group, and 8/10 participants in the OITA group passed the 8 g milk protein DBPCFC after maintenance period (*p* = 0.002). This trial has found that 6 of the 15 participants who passed 60-week DBPCFC lost desensitization to milk in less than 6 weeks. They did not completely lose desensitization to milk, and they were able to consume at least 2.5 oz of milk (at the beginning of trial it was just a teaspoon of milk). OIT was much more effective than SLIT, but with more systemic AEs.

In study of SLIT versus OIT for the treatment of peanut allergy results shown that pre-treatment with SLIT before OIT led to significant reduction in overall AE [77]. 

Kim et al. [72,73,74] conducted 3 significant clinical trials with SLIT in subjects with peanut allergy. All three studies included participants ages 1–11 years and they investigated efficacy and safety of SLIT for peanut allergy desensitization. The first study of SLIT treatment in children that have peanut allergy was the study of Kim et al. from 2011 [72]. It is a double-blind placebo RCT of 18 subjects with 6 months escalation plus 6 months of dose maintenance. During the DBPCFC median successfully consumed dose (SCD) for the treatment group was 1710 mg and for placebo group 85 mg, which represents significant difference between groups *p* = 0.011. These results represent potential protection from accidental peanut ingestion through everyday life situations and accidental exposure (often less than 100 mg peanut protein) [78]. The study by Kim et al. from 2019 was a RCT long-term SLIT for peanut allergy with 5 years total duration [73]. SLIT maintenance dose was 2 mg/d. Median SCD was 1750 mg peanut protein. After maintenance period and DBPCFC participants were without peanut protein exposure for 2–4 weeks and 10 of 12 participants reached SU. These results suggest that SLIT can enable safety through food exposure for most patients because tolerability of 300 mg peanut protein has 95% risk reduction [79] and in this study clinical threshold was greater than 1000 mg peanut protein. SU in this study was assessed after 2 to 4 weeks without any peanut exposure and that period might be too short for determination of real SU after therapy with SLIT. Another study from 2023 aimed to determinate the efficacy and safety of SLIT with 4 mg/d of peanut protein and durability of desensitization after SLIT. Their results suggest that SLIT can provide great results in desensitization even for a longer period without of exposure (more than 17 weeks) [74]. 

The first study from 2013 is a double-blind placebo RCT that lasted for 44 weeks (data available for 68 weeks), and the second study from 2015 is an open-label long-term follow-up study [75,76]. After 44 weeks of SLIT there was a statistically significant difference between intervention (14/20) and placebo group (3/20) *p* < 0.001. The study fulfilled its primary efficacy end point plus it showed great outcomes in those participants on higher maintenance dose (originally placebo group until week 44). Burks et al. [76] in their trial wanted to investigate the long-term (3-year) clinical and immunologic efficacy of peanut SLIT. This study showed modest desensitization and only 10.8% participants reached SU after SLIT therapy most likely because of a high participant dropout rate.

Immunological parameters were analysed in most of the reviewed studies, and they had statistical significance but none of them could reliably determine the best choice of immunotherapy or predict the level of therapy success.

### 4.2. Safety of SLIT

The majority of all AE in every trial were described as local oropharyngeal itching with no need for epinephrine use. SLIT is considerable as a very safe treatment with very good tolerance and very low rate of rate of side effects. 

All studies with SLIT and peanut allergy to date excluded patients with anaphylaxis history although it is extremely important to help such patients find an effective and safe treatment that would certainly have a positive effect on their quality of life.

## 5. Epicutaneous Immunotherapy (EPIT)

Epicutaneous immunotherapy (EPIT) is currently under investigation as a new type of immunotherapy for food allergy. Preclinical studies indicate that allergen applied via the epicutaneous route to intact skin does not cross into the circulation but rather activates dendritic cells in the dermal layer of the skin to affect immune activation [80]. EPIT is considered as a simple and safe method which does not interfere with everyday life and activities. Evidence shows that patients easily follow the immunotherapy protocol and tolerate this type of immunotherapy very well [81].

All studies reviewed in this paper used the Viaskin^®^ patch. Sweat effects on dissolution of the allergen in the patch and after the opening of skin pores allergen can be transferred to skin-based Langerhans cells with minimal risk of systemic absorption. Difference from other immunotherapy approaches is that EPIT delivers µg doses (rather than mg) of allergen, avoids oral route, and may have less AEs and better compliance than other types of immunotherapies [82]. The efficacy and safety of EPIT (in food allergy) with Viaskin patches has been investigated in several phase 2 and 3 controlled clinical trials, mostly on subjects with peanut allergy (Table 5).

### 5.1. Efficacy of EPIT

Prevalence of anaphylaxis and severe reactions to food in the paediatric population is the highest and most common in peanut food allergy [83]. It is very hard to avoid peanut consumption because of its widespread presence in various foods and dishes and unintentional exposure rates are high. There is a great need and longing for new and safe types of treatment for food allergies, especially peanuts so that patients and their families can have better overall quality of life [84]. It is estimated that most of peanut allergic children will have allergic reactions to < 1 peanut kernel (300 mg of peanut protein) [85]. So, one of the aims of this paper is to review efficacy and safety of EPIT as a treatment option in food allergy.

**Table 5:** Review of efficacy and safety in EPIT.

Author (Year)	Type of Study	Allergen	Participants	Duration	Efficacy	Safety
**Sampson et al. (2017) [81]** **VIPES**	multicentre double-blind placebo-RCT + 2-year, open-label extension study	peanut	n = 221 (6–55 y) randomization 1:1:1:1 Open Label Extension Study, n = 171	12 months + 2-year, open-label extension study	RR month 12 VP250-μg vs placebo *p* = 0.01; % responders only significant for the VP250 *p* = 0.04 RR in children VP250 vs. placebo *p* = 0.008; Open-label Extension study: RR at months 12 and 24 in the overall population 59.7% (89/149) and 64.5% (80/124)	AEs largely local skin reactions
**Jones et al. (2017) [86]** **CoFAR6**	Multicentre Double-blind placebo-RCT	peanut	n = 74 (4–25 y), median age 8.2 n = 25 placebo n_VP100_ = 24 or n_VP250_ = 25	52 weeks	Treatment success: VP100 vs. PLB *p* = 0.005;VP250 vs. PLB *p* = 0.003; VP100 vs. VP250, *p* = 0.48 -medium change SCD: Among 3 groups *p* = 0.003; Placebo vs. VP100 *p* = 0.014; Placebo vs. VP250 *p* = 0.003; VP100 vs. VP25 *p* = 0.41 -success better in younger participants (6–11 y) *p* = 0.006	AEs largely mild Non–patch-site AE: 0.2% of placebo and VP100 doses and 0.1% of VP250 doses
**Scurlock et al. (2021) [87] Follow-up CoFAR**	Open-label RCT	peanut	n = 74 (4–25 y)	130 weeks	Desensitization: 5% PLB-VP250, 20.8% VP100-VP250, 36% VP250 median SCD change from baseline of 11.5 mg, 141.5 mg, and 400 mg, respectively. Post-hoc analysis of change in SCD week 52–130: overall *p* = 0.29, within treatment groups PLB-VP250 *p* = 0.32; VP100-VP250 *p* = 0.32; VP250 *p* = 0.10.	most dosing AE mild
**Fleischer et al. (2019) [88]** **PEPITES**	multicentre Double-blind placebo-RCT	peanut	n = 356 (4–11 y) n = 238 peanut protein 250 μg; n = 118 placebo	12 months	The percentage difference in responders VP250-μg vs placebo *p* < 0.001 The lower bound of the 95% CI of the difference 12.4% crossed the prespecified lower limit of 15%	AE mostly mild 4 of 238 participants (1.7%) in the active group discontinued treatment due to AEs.
**Fleischer et al. (2020) [89]** **PEOPLE**	Open-label follow-up RCT	peanut	n = 198 (4–11)	5 years (4–5 y in progress)	141 (71%) subjects DBPCFC at month 36 At month 36: 51.8% subjects ED > 1000 mg, At month 12: 40.4%; 75.9% increased ED compared with baseline; 13.5% tolerated DBPCFC of 5444 mg. Median CRD from 144 to 944 mg; SU 14 of 18 subjects	AEs mild or moderate
**Greenhawt et al. (2023) [90]** **EPITOPE**	multicentre Double-blind placebo-RCT	peanut	n = 362 (1–3 y), the median age: 2.5 y intervention: placebo 244:118 ED < 300 mg	12 months	Intervention group 67.0% vs. placebo group 33.5%, *p* < 0.001) the mean change in CRD intervention vs placebo group 3.13 (*p* < 0.001) ED intervention vs placebo group 2.96 (*p* < 0.001)	AE mostly mild Serious AE 8.6% intervention group 2.5% placebo group; anaphylaxis 7.8% and 3.4%; Serious treatment-related AE 0.4% intervention group, no placebo group. Treatment-related anaphylaxis 1.6% intervention group, none placebo group.
**Dupont et al. (2010) [91]**	Double-blind placebo-RCT	Milk	n = 19 (3 months–15 y) n = 10 active group n = 9 placebo group	3 months	PP population CTD (0–90 day) *p* = 0.18	AE mild
**Spergel et al. (2020) [92]** **SMILEE**	pilot double- blind placebo- RCT (+ open-label extension study)	Milk	n = 20 (Age: 4–11 y) n = 15 active group, n = 5 placebo group	11 months + 11 months open label	VM500 group mean eos/hpf 50.1 ± 43.97 vs. the placebo group 48.20 ± 56.98 eos/hpf VM500 group lower mean eos/hpf count *p* = 0.038 Open label phase: 47% response mean values of fewer than 15 eos/hpf	Improvement in endoscopy scores in treatment group AE mild
**Pongracic et al. (2022) [93]** **REALISE**	multicentre Double-blind placebo-RCT + ongoing open-label active treatment	peanut	n = 393, (4–11 y) 3:1 = VP250:placebo for 6 months. 72.3% participants with history of peanut anaphylaxis	3 years (6 months double blind placebo control)	REALISE was without a DBPCFC and therefore had no efficacy assessment.	82.7% mild AE; 36.9% moderate AE 1.3% severe AE overall

RCT—randomized controlled trial; y—years; EPIT—epicutaneous immunotherapy; PP—per protocol; AE—adverse events; CTD—cumulative tolerated dose; VM500—Viaskin milk 500 µg; eos/hpf—eosinophils/high power fields; CRD—cumulative reactive dose; VP250—Viaskin peanut 250 µg; VP100—Viaskin peanut 100 µg; SCD—successfully consumed dose; RR—response rates; PLB—placebo; DBPCFC—double-blind placebo-controlled food challenge; ED—eliciting dose; SU—sustain unresponsiveness.

Efficacy represents the success of the implemented therapy, a treatment that safely and feasibly results in desensitization. The primary efficacy end point of most reviewed trials was the difference in response rates between intervention and placebo groups after a period (patients underwent a DBPCFC to establish changes in eliciting dose (ED)). The “Viaskin^®^ Peanut’s Efficacy and Safety” (VIPES) 2b double-blind placebo RCT looked at 3 doses of a peanut protein patch (50, 100, and 250 µg) doses across 221 subjects (6 to 55 years) at 22 centers for 12 months of treatment. After 12 months of treatment patients underwent a DBPCFC to establish changes in ED. Statistically significant difference in response rates (RR) was observed between the Viaskin peanut 250-μg (VP250) and placebo group *p* = 0.01. EPIT provided statistically better results in participants 6–11 years old with VP250 (*p* = 0.008) but not in participants older than 11 years [81]. Similar findings regarding age were found in the CoFAR6 study, also finding that participants younger than 11 years yield more benefit from EPIT than older participants [86].

CoFAR6 trial was conducted for 52 weeks and after that participant underwent to new open-label clinical trial for 130 weeks in total. In this study, all participants were in the VP250 group [87]. A total of 79.7% participants completed the 130 weeks of active treatment. This extended EPIT with VP250 was safe, well-tolerated with persistent desensitization during trial period. This study confirmed that treatment response was better in younger children (ages 4–11 years) receiving VP250. These three studies show better EPIT outcomes in younger participants which means that food allergy treatment should begin as early as possible to expect better treatment results.

Another randomized trial Peanut EPIT Efficacy and Safety (PEPITES) [88] used these findings and had 356 peanut-allergic children participants aged 4–11 years and used only VP250 patches on the intervention group.

The intervention group had significantly better treatment outcomes than the placebo group *p* < 0.001 but the lower bound of the 95% CI of the difference (12.4%) crossed the prespecified lower limit of 15%, and thus the trial could not be considered positive. Participants who completed PEPITES were offered enrolment in another study PEPITES Open-Label Extension (PEOPLE) [89]. In this study all participants were in the intervention group with VP250 for 24 or 36 months. After that period all participants had the opportunity to enrol in another 24-month treatment, totalling in 5 years of study in the same participants.

PEOPLE demonstrated durable efficient clinical outcomes of EPIT treatment in children with peanut allergy (4–11 years old). Results of PEOPLE showed persistent desensitization to allergen with EPIT treatment over longer periods of time. After 36 months ¾ participants had better ED compared with the beginning of the trial and more than 1/2 of participants at 36 months had ED of > 1000 mg. Those participants with ED > 1000 mg underwent complete elimination of peanut for 2 months to determine a SU. SU was reached in 77.8% of participants (14 of 18). Conclusions about EPIT efficacy in establishment of SU cannot be made because of a small number of people that could participate in the SU assessment.

A recently published RCT (EPITOPE) [90] made a step forward in efficiency research of EPIT in peanut allergic children age range 1–3 years. The primary efficacy end point in the active group was significant greater compared with the placebo group (*p* < 0.001). Participant in the active VP250 group had a median change in ED (from start of the trial to month 12) of 900 mg vs. 0 mg in the placebo group participants (*p* < 0.001). This showed that EPIT can be a useful tool in treatment of peanut allergy in children younger than 4 years. Open-label extension of the EPITOPE trial is ongoing, and we hope that those results will provide significant information regarding the efficacy of EPIT and its potential use in everyday clinical work. 

Children with peanut allergy have high rates of comorbidities such as atopic dermatitis, asthma, or other allergies especially food allergies. Davis et al. [94] conducted the study (data from PEPITES and REALISE study) of the safety and efficacy of EPIT in peanut-allergic children with these comorbidities. Peanut EPIT showed statistically significant efficiency of the therapy, regardless of the mentioned comorbidities. 

There are a few clinical trials related to EPIT and other food allergens that are available. Here we will highlight studies that show promising results regarding EPIT and effectiveness of the method.

A pilot study from 2010 evaluates the safety of EPIT in children (age range 3 months–15 years) for 3 months under therapy (active or placebo). Active patches contained 1 mg skimmed cow’s milk powder. In this preliminary study cumulative tolerated dose (CTD) failed to demonstrate a statistically significant improvement, most likely due to very the short duration of the trial. Results suggested that EPIT with milk protein is well-tolerated [91].

SMILEE [92] is an RCT study with aim to determine efficacy and safety of EPIT with Viaskin milk (500 µg milk proteins, VM500) in children with milk induced EoE. Seven participants that were on VM500 treatment vs. 2 placebo participants had a significantly lower mean eos/hpf count *p* = 0.038. At the end of the open-label phase 47% participants had mean values of fewer than 15 eos/hpf which means that EPIT might not be effective just for IgE-mediated food allergy but also for non-IgE-mediated food allergies.

All clinical trials reviewed in this paper indicate that EPIT has promising effect in desensitization and that it can be a useful tool for treatment food allergy.

### 5.2. Safety of EPIT

EPIT is considered as a safe treatment, especially compared with other types of immunotherapies. All studies reviewed in this paper highlight EPIT as a highly safe method. Symptoms are mostly application site related, and mostly mild to moderate [81,86,87,88,89,90,91,92,93]. Adherence in most studies is very good, except SMILEE [92] where a high rate of protocol violations was reported (nonadherence to diet therapy, noncompliance with PPI dosing). 

The only trial that included participants with a history of anaphylaxis and exclude DBPCFC as a part of trial procedure is REALISE (Real Life Use and Safety of EPIT) RCT [93] which provides more reliable results in regards of safety, closer to real life for people with food allergy. Participants were highly atopic and 72.3% participants had a history of anaphylaxis to peanut protein. Compliance to treatment was very high, most of reported AEs were mild (82.7%) or moderate (36.9%). Findings from REALISE suggest that EPIT is a safe and well-tolerated method of immunotherapy for food allergy regardless of the possibility of developing severe symptoms and anaphylaxis. Given all the above, it can be concluded that EPIT has a potential impact on substantial reduction in allergic reactions to peanut. These findings are essential for people with food allergies and their families, especially in regards of improving their quality of life. This could also lay a basis for the implementation of food allergy related immunotherapy in routine clinical use.

## 6. Food Allergen Immunotherapy and Biologics

There are many novel therapeutic approaches being investigated for the treatment of food allergy such as micobiome modulating drugs and biologicals. Biologicals are promising therapeutics which target the underlying immune response driving food allergy. Among these biological drugs, omalizumab, or anti-IgE antibody, is most commonly used, both in clinical studies and in clinical practice, as monotherapy in patients with severe IgE-mediated food allergy or in combination with FAIT [95,96]. It has shown favourable effects for improving the safety and efficacy of OIT as well as its potential use in the management of food allergies independently of OIT. Therapy with omalizumab increases the threshold of reactivity to foods and increases the tolerated dose of foods when given as monotherapy or in combination with OIT. Omalizumab administered during the up-dosing phase of OIT shortens the time required to reach the maintenance dose. Furthermore, omalizumab can also prevent systemic allergic reactions including anaphylaxis when given as an adjunct to immunotherapy [95,96,97]. According to the above, omalizumab is used in clinical practice today as a key and irreplaceable therapy for reducing the risks associated with FAIT, especially in patients with risk of severe anaphylactic reactions, or sensitized to very low doses of food allergen or allergen components, as well as in treatment of patients with multiple food allergies or patients with comorbidities, such as allergic asthma [98,99,100].

Ibáñez-Sandín et al. [101] analysed the effect of omalizumab therapy as monotherapy or in combination with OIT in the treatment of patients with severe cow’s milk allergy under real-life conditions. Omalizumab which is used as monotherapy induced tolerance to ≥6000 mg of cow’s milk protein in 34.8% of patients, while in combination with milk OIT, up to 83.0% of patients has achieved tolerance to cow’s milk proteins. They also analysed the safety effect of omalizumab and observed that stopping omalizumab therapy significantly increases the risk of AE including anaphylaxis (in 36.4% of patients, *p* = 0.001) compared with patients who received continuous omalizumab during OIT. In patients who abruptly discontinued omalizumab anaphylaxis was observed in 50.0%, and only in 12% of patients who gradually discontinued oamlizumab therapy during OIT.

There are many other upcoming biologicals that are currently under investigation in ongoing clinical trials such as the anti-IL4 receptor α antibody or dupilumab, and ligelizumab or the next generation of anti-IgE antibody, and tezepelumab that blocks the activity of TSLP and etokimab or the anti-interleukin 33 antibody [100,102]. Although there are more and more publications on the use of different biological drugs in the treatment of patients with food allergy, both as monotherapy and in combination with FAIT biological agents have not yet been approved in the treatment of food allergies in clinical practice [100,103,104].

Different biologics have different effects on the modulation of the immune response in the treatment of patients with food allergy directly targeting the specific underlying mechanism. This is precisely why an individualized approach is key for future research which should be focused on personalized criteria for patient selection, discovery of biomarker panels, therapy duration and proper dosing, monitoring of efficacy, etc.

## 7. Discussion

In the last 10 years, the approach to the management and treatment of food allergy has significantly changed from a preventive-symptomatic approach to etiological treatment. The introduction of FAIT both oral, and other modifications, such as SLIT and EPIT, with an overview of efficacy and safety for the most frequently applied nutritional allergens (cow’s milk, eggs, and peanuts) are shown in this article. In the 2018 European Academy of Allergy and Clinical Immunology (EAACI) published guidelines on AIT recommending the application of OIT in specialized centres with expertise in AIT and management of anaphylactic shock [1]. The US Food and Drug Administration (FDA) approved Pelforzia in January 2020, a drug for peanut OIT in children 4 to 17 years old with the indication of “the mitigation of allergic reactions, including anaphylaxis that may occur with accidental exposure to peanut” [4,5]. Although many real-life studies reported an acceptable ratio of OIT safety and efficacy using commercial standardized food product, there is no evidence on the superiority of use pharmaceutical OIT products over non-standardized conventional. food products. In addition, weak point of commercial standardized food products could be a high price which could limit access to OIT treatment [71].

Despite different protocols, undefined thresholds of tolerance, partial effectiveness in reaching permanent and SU as well as safety limitations of OIT to milk, egg, and peanut is increasingly applied in clinical practice [1]. At the beginning OIT may have higher cost compared with avoidance, which are associated mainly with often physician visits, but on maintenance will be reduced and possibly lower than avoidance. Therefore, long-term OIT may be cheaper than avoidance for patient family, but also for healthcare system [41].

A significant improvement in the quality of life of patients who have raised the tolerance threshold and significantly reduced the risk of anaphylaxis after accidental exposure to small doses of food allergens, can be achieved in almost all treated patients.

De facto, patients with severe food allergy which react to very low doses may fail to reach the maintenance-dose of OIT with high risk of adverse events or anaphylaxis. For such patients, the goal should be low-dose OIT, treating them with a very gradual increase in doses, with the aim of reaching a threshold that will protect them from accidental exposure to foods containing traces of the allergen with an acceptable safety profile during OIT.

Yanagida N et al. [105] reported low-dose OIT studies of patients with severe milk allergy. They consumed 3 mL of heated milk or 10 g of butter daily for 1 year. OFC was performed by using 25 mL of heated milk or 15 g of boiled noodles. About 50% of patients who were on a low-dose OIT in the challenge test did not develop an allergic reaction, with significantly fewer side effects in comparison with the group that was on conventional OIT. Low-dose OIT may be a favourable option for patients with severe allergic reactions caused by food allergens.

Unlike OIT which is widely used with allergens that are not commercially available and standardized, SLIT for food allergy involves placement of glycerinated allergen under the tongue daily. SLIT has been researched in the treatment of milk, peanut, hazelnut, peach, and apple allergies. Peanut allergy treatment is the most thoroughly studied application of food SLIT, as we as we have previously shown. The studies have shown that SLIT had fewer and less severe side effects than other modalities, but the achieved maintenance dose was significantly lower than in patients on OIT [71]. SLIT was not as effective as OIT in achieving desensitization to cow’s milk but was associated with fewer side-effects, they are most often local, limited to the oropharynx. 

Future directions for SLIT are under development with the aim of increasing the daily dose applied orally without increasing side effects [36]. A novel delivery platform named as oral mucosal immunotherapy (OMIT) goals to deliver allergen to the oral mucosa with the highest concentration of oral Langerhans cells [106]. 

Several advantages have been recognized for EPIT: (a) provides a high safety profile due to method of allergen application, (b) increase in the convenience for the patients due to non-invasive (needle-free) application, (c) improved compliance and self-administrable application (d) free of irritant constituents (e.g., preservatives, aluminium), (e) and acceptable price. Currently, the effectiveness of EPIT has been published in a limited number of clinical studies and has been investigated on the small number of food allergens (most often on peanuts) (Table 5). Consequently, the long-term efficacy of EPIT regarding sustained tolerance has not been published so far. Pre-treatment of the skin during EPIT enhances the permeability which stimulate allergen delivery to the immune cells. For safety and efficacy of EPIT the disruption of the skin-barrier seems crucial (e.g., skin integrity, pH, hydration, temperature, inflammation as well as the position of patch application). Efficacy of EPIT (using Viaskin^®^ technology) has been primarily demonstrated for children [81,92]. Further studies are necessary to optimize the treatment regime (e.g., allergen dose, pre-treatment modalities, number, and interval of patch application, etc.) for better balance between efficacy and safety. 

## 8. Conclusions

FAIT is the only disease-modifying treatment option for individuals with IgE-mediated food allergy. It has been shown that FAIT is a clinically effective and safe treatment option for patients with clinically relevant food allergy. Although FAIT is generally an effective treatment option, some patients do not respond well. The understanding of underlying mechanisms in FAIT is lacking, but most are related to the modulation of the innate and adaptive immune responses, which are also related to the effectiveness of the treatment. There has been no clear relationship between the immunological changes observed and the level of response to FAIT. Further research is needed to confirm and interpret these associations with different route, doses, duration, frequency of application and clinical response to FAIT. On the other hand, the occurrence offside effects, including anaphylactic reactions during OIT are significant, and further narrow the indications for use, which calls for additional research as well.

Future perspectives in the field of FAIT and food allergy management include larger, multi-centre DBPC RCTs, new and improved protocols, extending the age limitations for administering FAIT, inclusion of different allergens and allergen components and finally, novel routes of administration (such as OMIT).

## Figures and Tables

**Table 1:** Review of efficacy and safety of OIT to milk.

Reference	Design	Sample	Participants Characteristic	Form of Allergen	Duration	Maintenance Dose	Efficacy	Safety
**milk**								
**Skripak et al. (2008) [40]**	Double-blind, placebo-RCT	n = 20 (6–17 y) OIT: n = 12 Placebo: n = 7	Positive DBPCFC to 2.5 g MP. Baseline median threshold: 40 mg MP	Milk powder	5–6 months	500 mg MP (15 mL of milk)	Median threshold after OIT: OIT: 5140 mg Placebo: 40 mg (*p* = 0.003)	AE/total doses: OIT 45.4%, placebo: 11.2% AE/each participant: OIT 35%, placebo group 1%. AAR: OIT 1%, placebo 0%
**Narisety et al. (2009) [41]**	Extension of previous *p* study [40]	n = 15 (6–16 y)	Negative DBPCFC to 2.5 g MP	Dairy products	3–17 months	Daily diary intake at home	OFC to 16 g MP: Negative for 33%	AE/total doses: 17% Epinephrine: 0.2% reaction
**Inuo et al. (2018) [46]**	Double-blind, placebo-RCT, 2 phase: 8 weeks pHF or eHF, then 8 weeks all on eHF	n= 25 (1–9 y) 2 group: active: pHF-pHF (n = 13) placebo: eHF-eHF (n = 12)	History of systemic reactions to milk. Positive OFC to 20 mL rCMF	pHF	16 weeks	0.5–20 mL of pHF	OFC with rCMF: Threshold at the end of first phase: Significantly elevated in pHF.pHF (*p* = 0.048)	AE: not severe reaction 2 participants in active group mild reaction
**Nagakura et al. (2021) [44]**	Open-label RCT	n = 33, >5 y HM-OIT: (n = 17) UM-OIT (n = 16)	Positive DBPCFC on 3-mL HM, History of milk anaphylaxis	HM vs. UM	1 year	3 mL milk	Desensitization to 3 mL and 25 mL: HM-OIT: 35%, 18% UM-OIT; 50%, 31% (*p* = 0.34, *p* = 0.43)	AE at home dose: HM: OIT: 8.1%; UM-OIT: 9.6% AE moderate/severe at home: HM-OIT: 0.7%; UM-OIT: 1.4% (*p* = 0.0002)
**Maeda et al. (2021) [42]**	Open-label RCT	n = 28, 3–12 y OIT: n = 14 Control: n = 14	Positive OFC to 10 mL milk	Liquid milk	1 year	100 mL milk	OFC to 100 mL milk: OIT: 50% Control: 0% (*p* < 0.01)	AE requiring adrenaline: OIT: 43% participants Control: 0% participants
**Van Boven et al. (2023) [45]**	RCT follow-up study	n = 18 (6–36 months) OIT: n = 11 control: n = 7	Milk allergy diagnosed by allergist)	iAGE	24 months	5% total protein intake/day	DBPCFC (4.3 g MP): OIT vs. placebo: T1 (8 months); 73 vs. 57% T3 (24 months): 82 vs. 71%	AE: no product related

RCT—randomized controlled trial; y—years; OIT—oral immunotherapy; DBPCFC—doble-blind placebo-controlled food challenge; MP—milk protein; AE—adverse events; anaphylactic adverse reactions; OFC—oral fool challenge; pHF—partially hydrolyzed formula; eHF—extensively hydrolyzed formula; rCMF—regular caw’s milk formula; HM—heated milk; UM—unheated milk; iAGE—heated milk protein standard product.

**Table 2:** Review of efficacy and safety of OIT to egg.

Reference	Design	Sample	Participants Characteristic	Form of Allergen	Duration	Maintenance Dose	Efficacy	Safety
**Burks et al. (2012) [47]**	Double-blind, placebo-RCT	n = 55 (5–11 y) OIT: 40 Placebo: 15	Clinical history of egg-allergy	EWP	24 months	2 g EWP	At 10 and 22 months: Desensitization to 5 and 10 g of EPW: OIT: 55% and 75% Placebo: 0% and 0% (*p* < 0.00, *p* < 0.0011) At 24 months: SU to 10 g EPW + whole cooked egg: OIT: 11%, placebo: 0% (*p* < 0.03)	AE: % participants OIT: 78%; Placebo: 20% (*p* < 0.01) Severe AE: no
**Jones et al. (2016) [7]**	Extension of previous study [47] at 4 y	n = 55 (5–11 y) OIT: 40 Placebo: 15	Clinical history of egg-allergy	EWP	2 y	2 g EWP	SU to 10 g EPW + whole cooked egg by 3 and 4 y: OIT: 45% and 50%	AE during OIT dosing: 54% OIT participants, mostly mild symptoms
**Itoh-Nogato et al. (2018) [50]**	Randomized, parallel-group, delayed-start study. 1st stage: early start group on rush OIT. 2nd stage: all participants on OIT	n = 45 (5–15 y) early start: n = 23 (received rush OIT for 3 months) late-group: n = 22 (continue egg elimination for 3 months before OIT)	positive DBPCFC to ≤500 mg dried raw EPW	EWP	1 y	60 g of cooked egg ~1 medium size egg or 1 g EWP	Desensitization to 1000 mg EPW after 3 months: Early star: 87% Late start: 22% (*p* < 0.001).	AE during first stage: Early start: 80% Late-star: 0% AE requiring adrenaline; 11.6%
**Kim et al. (2020) [48]**	Extension of previous published study [47] at 5 y	n = 55 (5–11 y) OIT: 40 Placebo: 15	Clinical history of egg-allergy, Completed previous OIT study		1 y	Unlimited consumption all form of egg	Ingestion all form of egg: SU-OIT: 100% Desensitized OIT: 43% Non-desensitized OIT: 17% Placebo: 36%	AE: no OIT participants reported symptoms to any baked egg consumption
**Kim et al. (2020) [6]**	Open label randomized trial	n = 50 (3–36 y) BE-R: n = 27 OIT-R: n = 23. OIT-assigned (OIT-A) comparison: 39	Negative DBPCFC to BE Positive DBPCFC to unbaked egg (1444 mg of EWP)	BE vs. EWP	2 y	2000 mg egg white protein	SU to 7444 mg white egg protein: BE-R = 11.1% OIT-R: 43.15% OIT-A 17.9%	AE: % participants: BE-R: 2.8% OIT-R: 3.9% OIT-A: 12.6% Severe AE: only in OIT groups
**Palosuo et al. (2021) [49]**	Open-label randomized trial	n = 50 (6–17 y) OIT: 32 Control: 18	Positive DBPCFC to heated egg white	EWP	8 months	1 g egg-white protein	Desensitization to 1 g of egg white protein: OIT: 44% Control: 4.8%	AE: 82% participants during build-up phase No severe reactions

RCT—randomized controlled trial; y—years; OIT—oral immunotherapy; EWP—egg white powder; SU—sustain unresponsiveness; AE—adverse events; DBPCFC—double-blind placebo-controlled food challenge; BE-R—baked egg randomized; OIT-R—OIT randomized; OIT-A—OIT assigned.

**Table 3:** Review of efficacy and safety of OIT to peanut.

Reference	Design	Sample	Participants Characteristic	Form of Allergen	Duration	Maintenance Dose	Efficacy	Safety
**Varshney et al. (2011) [56]**	Double-blind placebo RCT	n = 25 (1–16 y) OIT = 16 Placebo = 9	Clinical history of reaction to peanut (<60 min after ingestion)	Peanut flour	1 y	4000 mg PP ~15–16 peanuts	Desensitization to 5 g PP: OFC: 100%, placebo: 0%	AE/dose: 1.2% in OIT participants during build-up phase Epinephrine: no
**Anagnostou et al. (2014) [57]**	Crossover 2-phase-RCT (STOP II)	n = 85 (7–16 y) OIT: 46 Control: 39 2.phase: Control -> OIT	Immediate reaction after peanut ingestion, positive DBPCFC	Peanut flour	6 months	800 mg PP	Desensitization to 1400 mg PP: First phase: OIT: 62%, control: 0% (*p* < 0.001) Second phase: Control after OIT: 54%	AE per OIT dose: 6.3%- mild reaction Adrenaline: 0.01% dose
**Vickery et al. (2017) [58]**	Double-blind placebo RCT	n = 37 (9–36 months) Low dose (LD): 20 High dose (HD): 17	Positive OFC to 4 g PP	Peanut-flour	22–36 months	PP: LD: 300 mg HD: 3000 mg	SU to 5 g PP: 29/37 (78%) LD: 85%; HD: 71%, *p* = 0.43 Control: 4%	AE: % participants: LD: 90%, HD 100%
**Bird et al. (2017) [60]**	Double-blind placebo RCT Phase 2 (ARC001)	n = 55 (4–26 y) AR101: 29 Placebo: 26	Positive DBPCFC to 143 mg PP	AR101-comercial product	20–34 weeks	300 mg PP	Desensitization to 300 mg PP AR101: 79%, placebo: 19% (*p* < 0.001) Desensitization to 600 mg PP: AR101: 62%, placebo: 0% (*p* < 0.001)	AE: % participants during treatment: AR101: 93%, placebo: 46%. No severe AE
**Vickery et al. (2018) [61]**	Double-blind placebo-RCT Phase 3 (PALISADE)	n = 496 (4–17 y) AR101: 372 Placebo: 124	Positive DBPCFC to 100 mg PP (1/3 peanut)	AR101-comercial product	1 y	300 mg PP	Desensitization to 600 mg PP: AR101: 67.2%, placebo: 4% (*p* < 0.001)	AE: % participants: AR101: 98.7%, placebo: 95.2% Severe AR: AR101: 4.3%, placebo: 0.8%
**Chintrajah et al. (2019) [33]**	Double-blind placebo-RCT 2 phase study Peanut-0: no peanut after OIT Peanut-300: 300 mg PP daily after OIT Placebo: received placebo (POISED study)	n = 120 (7–55 y) Peanut-0: 60 Peanut-300: 35 placebo: 25	Positive DBPCFC to 500 mg PP	Peanut flour	3 y	4 mg od PP	Negative DBPCFC to 4 g PP at week 104 (desensitization) and 117 (SU): Peanut-0: 85%, 35% Peanut-300: 83%, 54% Placebo: 4%, 4% SU to 4 g PP at week 156: Peanut-0: 13 Peanut-300: 37%	AE: % participants through 1st to 3rd years: Peanut-0: 95–2% Peanut-300: 91–20% Placebo: 64–5%
**Vickery et al. (2020) [62]**	Open-label follow-on study of previous study [61] (ARC004) PTAH = formerly AR101	n = 358 (4–17 y) PTAH: 256 (A) PTAH: daily dosing: 300 mg daily (B) PTAH: non- daily dosing 300 mg (e.g., biweekly) (C) PTAH-naive: 102	PTAH: negative DBPCFC to 300 mg PP PTAH naïve: placebo arm from PALISADE	PTAH (Palforzia commercial product)	1–2 y	300 mg of PTAH	Desensitization to 2 g PP Daily dosing > non-daily dosing	AE: almost all PTAH participants Daily dosing < non-daily dosing
**Jones et al. (2022) [59]**	Double-blind placebo RCT (IMPACT trial)	n = 146 (12–48 months) OIT: 96 Placebo = 50	Positive DBPCFC to 500 mg PP	Peanut flour	160 weeks	2000 mg PP	Desensitization to 5 g PP OIT: 71%, placebo: 2% (*p* < 0.0001) SU to 5 g PP: OIT: 21%, placebo: 2% (*p* = 0.0021)	AE: % participants: OIT: 98%, placebo: 80% Epinephrine: OIT 22%, placebo 0%
**Fernandez-Rivas et al. (2022) [63]**	Open label follow-on study of previous study [62]	n = 130 (4–17 y) A: 104 (1.5 y) B: 26 (2 y)	negative DBPCFC to 300 mg PP	PTAH (Palforzia- commercial product)	1.5–2 y	300 mg of PTAH	DBPCFC to 2 g PP: A: 48.1%; B: 80.8%	AE: decreased throughout the intervention period in both groups

RCT—randomized controlled trial; y—years; OIT—oral immunotherapy; PP—peanut protein; AE—adverse events; DBPCFC—double-blind placebo-controlled food challenge; LD—low dose; HD—high dose; SU—sustain unresponsiveness; PTAH—peanut (*Arachis hypogaea*) allergen powder-dnfp (Palforzia^®^, Aimmune Therapeutic.

## References

[1] Pajno G.B., Fernandez-Rivas M., Arasi S., Roberts G., Akdis C.A., Alvaro-Lozano M., Beyer K., Bindslev-Jensen C., Burks W., Ebisawa M. (2018). EAACI Allergen Immunotherapy Guidelines Group. EAACI Guidelines on allergen immunotherapy: IgE-mediated food allergy. Allergy.

[2] Alvaro-Lozano M., Akdis C.A., Akdis M., Alviani C., Angier E., Arasi S., Arzt-Gradwohl L., Barber D., Bazire R., Cavkaytar O. (2020). Allergen Immunotherapy User’s Guide. Pediatr. Allergy Immunol..

[3] Muraro A., de Silva D., Halken S., Worm M., Khaleva E., Arasi S., Dunn-Galvin A., Nwaru B.I., De Jong N.W., Rodríguez Del Río P. (2022). GA2LEN Food Allergy Guideline Group; GALEN Food Allergy Guideline Group. Managing food allergy: GA2LEN guideline 2022. World Allergy Organ. J..

[4] Hise K., Rabin R.L. (2020). Oral Immunotherapy for Food Allergy-a US Regulatory Perspective. Curr. Allergy Asthma Rep..

[5] Leonard S.A., Laubach S., Wang J. (2021). Integrating oral immunotherapy into clinical practice. J. Allergy Clin. Immunol..

[6] Kim E.H., Perry T.T., Wood R.A., Leung D.Y.M., Berin M.C., Burks A.W., Cho C.B., Jones S.M., Scurlock A., Consortium for Food Allergy Research (CoFAR) (2020). Induction of sustained unresponsiveness after egg oral immunotherapy compared to baked egg therapy in children with egg allergy. J. Allergy Clin. Immunol..

[7] Jones S.M., Burks A.W., Keet C., Vickery B.P., Scurlock A.M., Wood R.A., Liu A.H., Sicherer S.H., Henning A.K., Lindblad R.W. (2016). Long-term treatment with egg oral immunotherapy enhances sustained unresponsiveness that persists after cessation of therapy. J. Allergy Clin. Immunol..

[8] Sampath V., Sindher S.B., Alvarez Pinzon A.M., Nadeau K.C. (2020). Can Food Allergy be Cured? What are the Future Prospects?. Allergy.

[9] Du Toit G., Roberts G., Sayre P.H., Bahnson H.T., Radulovic S., Santos A.F., Brough H.A., Phippard D., Basting M., Feeney M. (2015). LEAP Study Team. Randomized trial of peanut consumption in infants at risk for peanut allergy. N. Engl. J. Med..

[10] Akdis C.A., Akdis M. (2015). Mechanisms of allergen-specific immunotherapy and immune tolerance to allergens. World Allergy Organ. J..

[11] Kulis M.D., Patil S.U., Wambre E., Vickery B.P. (2018). Immune mechanisms of oral immunotherapy. J. Allergy Clin. Immunol..

[12] Schoos A.M., Bullens D., Chawes B.L., Costa J., De Vlieger L., DunnGalvin A., Epstein M.M., Garssen J., Hilger C., Knipping K. (2020). Immunological Outcomes of Allergen-Specific Immunotherapy in Food Allergy. Front. Immunol..

[13] Monian B., Tu A.A., Ruiter B., Morgan D.M., Petrossian P.M., Smith N.P., Gierahn T.M., Ginder J.H., Shreffler W.G., Love J.C. (2020). Peanut Oral Immunotherapy Suppresses Clonally Distinct Subsets of T Helper Cells. SSRN Electron. J..

[14] Wawrzyniak M., O’Mahony L., Akdis M. (2017). Role of regulatory cells in oral tolerance. Allergy Asthma Immunol. Res..

[15] Baloh C.H., Huffaker M.F., Laidlaw T. (2022). Biomarkers and mechanisms of tolerance induction in food allergic patients drive new therapeutic approaches. Front. Immunol..

[16] Ponsonby A.L., Collier F., O’Hely M., Tang M.L.K., Ranganathan S., Gray L., Morwitch E., Saffery R., Burgner D., Dwyer T. (2022). BIS Investigator Group. Household size, T regulatory cell development, and early allergic disease: A birth cohort study. Pediatr. Allergy Immunol..

[17] Syed A., Garcia M.A., Lyu S.C., Bucayu R., Kohli A., Ishida S., Berglund J.P., Tsai M., Maecker H., O’Riordan G. (2014). Peanut oral immunotherapy results in increased antigen-induced regulatory T-cell function and hypomethylation of forkhead box protein 3 (FOXP3). J. Allergy Clin. Immunol..

[18] Vardar Acar N., Cavkaytar Ö., Arik Yilmaz E., Büyüktiryaki A.B., Uysal Soyer Ö., Şahiner Ü.M., Şekerel B.E., Karaaslan I.Ç., Saçkesen C. (2020). Rare occurrence of common filaggrin mutations in Turkish children with food allergy and atopic dermatitis. Turk. J. Med. Sci..

[19] Bajzik V., DeBerg H.A., Garabatos N., Rust B.J., Obrien K.K., Nguyen Q.A., O’Rourke C., Smith A., Walker A.H., Quinn C. (2022). Oral desensitization therapy for peanut allergy induces dynamic changes in peanut-specific immune responses. Allergy.

[20] Berin M.C., Agashe C., Burks A.W., Chiang D., Davidson W.F., Dawson P., Grishin A., Henning A.K., Jones S.M., Kim E.H. (2022). Allergen-specific T cells and clinical features of food allergy: Lessons from CoFAR immunotherapy cohorts. J. Allergy Clin. Immunol..

[21] Tsai M., Mukai K., Chinthrajah R.S., Nadeau K.C., Galli S.J. (2020). Sustained successful peanut oral immunotherapy associated with low basophil activation and peanut-specific IgE. J. Allergy Clin. Immunol..

[22] Kulis M., Yue X., Guo R., Zhang H., Orgel K., Ye P., Li Q., Liu Y., Kim E., Burks A.W. (2019). High- and low-dose oral immunotherapy similarly suppress pro-allergic cytokines and basophil activation in young children. Clin. Exp. Allergy.

[23] Patil S.U., Steinbrecher J., Calatroni A., Smith N., Ma A., Ruiter B., Virkud Y., Schneider M., Shreffler W.G. (2019). Early decrease in basophil sensitivity to Ara h 2 precedes sustained unresponsiveness after peanut oral immunotherapy. J. Allergy Clin. Immunol..

[24] Gorelik M., Narisety S.D., Guerrerio A.L., Chichester K.L., Keet C.A., Bieneman A.P., Hamilton R.G., Wood R.A., Schroeder J.T., Frischmeyer-Guerrerio P.A. (2015). Suppression of the immunologic response to peanut during immunotherapy is often transient. J. Allergy Clin. Immunol..

[25] Patil S.U., Ogunniyi A.O., Calatroni A., Tadigotla V.R., Ruiter B., Ma A., Moon J., Love J.C., Shreffler W.G. (2015). Peanut oral immunotherapy transiently expands circulating Ara h 2-specific B cells with a homologous repertoire in unrelated subjects. J. Allergy Clin. Immunol..

[26] Vickery B.P., Scurlock A.M., Kulis M., Steele P.H., Kamilaris J., Berglund J.P., Burk C., Hiegel A., Carlisle S., Christie L. (2014). Sustained unresponsiveness to peanut in subjects who have completed peanut oral immunotherapy. J. Allergy Clin. Immunol..

[27] Kulis M., Saba K., Kim E.H., Bird J.A., Kamilaris N., Vickery B.P., Staats H., Burks A.W. (2012). Increased peanut-specific IgA levels in saliva correlate with food challenge outcomes after peanut sublingual immunotherapy. J. Allergy Clin. Immunol..

[28] Pier J., Liu E.G., Eisenbarth S., Järvinen K.M. (2021). The role of immunoglobulin A in oral tolerance and food allergy. Ann. Allergy Asthma Immunol..

[29] Kulis M., Smeekens J., Larson D., Qin T., Burks A.W. (2020). Peanut-Specific IgA and IgG4 in Saliva are Modulated by Peanut OIT. J. Allergy Clin. Immunol..

[30] Santos A.F., James L.K., Kwok M., McKendry R.T., Anagnostou K., Clark A.T., Lack G. (2020). Peanut oral immunotherapy induces blocking antibodies but does not change the functional characteristics of peanut specific IgE. J. Allergy Clin. Immunol..

[31] van de Veen W., Stanic B., Yaman G., Wawrzyniak M., Söllner S., Akdis D.G., Rückert B., Akdis C.A., Akdis M. (2013). IgG4 production is confined to human IL-10-producing regulatory B cells that suppress antigen-specific immune responses. J. Allergy Clin. Immunol..

[32] Takasato Y., Kurashima Y., Kiuchi M., Hirahara K., Murasaki S., Arai F., Izawa K., Kaitani A., Shimada K., Saito Y. (2021). Orally desensitized mast cells form a regulatory network with Treg cells for the control of food allergy. Mucosal Immunol..

[33] Chinthrajah R.S., Purington N., Andorf S., Long A., O’Laughlin K.L., Lyu S.C., Manohar M., Boyd S.D., Tibshirani R., Maecker H. (2019). Sustained outcomes in oral immunotherapy for peanut allergy (POISED study): A large, randomised, double-blind, placebo-controlled, phase 2 study. Lancet.

[34] Anvari S., Watkin L.B., Minard C.G., Schuster K., Hassan O., Anagnostou A., Orange J.S., Corry D.B., Davis C.M. (2022). Reduced pro-inflammatory dendritic cell phenotypes are a potential indicator of successful peanut oral immunotherapy. PLoS ONE.

[35] Allam J.P., Würtzen P.A., Reinartz M., Winter J., Vrtala S., Chen K.W., Valenta R., Wenghoefer M., Appel T., Gros E. (2010). Phl p 5 resorption in human oral mucosa leads to dose-dependent and time-dependent allergen binding by oral mucosal Langerhans cells, attenuates their maturation and enhances their migratory and TGF-β1 and IL-10–producing properties. J. Allergy Clin. Immunol..

[36] Schworer S.A., Kim E.H. (2020). Sublingual immunotherapy for food allergy and its future directions. Immunotherapy.

[37] Scheurer S., Toda M. (2017). Epicutaneous immunotherapy. Allergol. Immunopathol..

[38] Bégin P., Chan E.S., Kim H., Wagner M., Cellier M.S., Favron-Godbout C., Abrams E.M., Ben-Shoshan M., Cameron S.B., Carr S. (2020). CSACI guidelines for the ethical, evidence-based and patient-oriented clinical practice of oral immunotherapy in IgE-mediated food allergy. Allergy Asthma Clin. Immunol..

[39] Begin P., Chinthrajah R.S., Nadeau K.C. (2014). Oral immunotherapy for the treatment of food allergy. Hum. Vaccin. Immunother..

[40] Skripak J.M., Nash S.D., Rowley H., Brereton N.H., Oh S., Hamilton R.G., Matsui E.C., Burks A.W., Wood R.A. (2008). A randomized, double-blind, placebo-controlled study of milk oral immunotherapy for cow’s milk allergy. J. Allergy Clin. Immunol..

[41] Narisety S.D., Skripak J.M., Steele P., Hamilton R.G., Matsui E.C., Burks A.W., Wood R.A. (2009). Open-label maintenance after milk oral immunotherapy for IgE-mediated cow’s milk allergy. J. Allergy Clin. Immunol..

[42] Maeda M., Imai T., Ishikawa R., Nakamura T., Kamiya T., Kimura A., Fujita S., Akashi K., Tada H., Morita H. (2021). Effect of oral immunotherapy in children with milk allergy: The ORIMA study. Allergol. Int..

[43] Geiselhart S., Podzhilkova A., Hoffmann-Sommergruber K. (2021). Cow’s Milk Processing-Friend or Foe in Food Allergy?. Foods.

[44] Nagakura K.I., Sato S., Miura Y., Nishino M., Takahashi K., Asaumi T., Ogura K., Ebisawa M., Yanagida N. (2021). A randomized trial of oral immunotherapy for pediatric cow’s milk-induced anaphylaxis: Heated vs. unheated milk. Pediatr. Allergy Immunol..

[45] van Boven F.E., Arends N.J.T., Sprikkelman A.B., Emons J.A.M., Hendriks A.I., van Splunter M., Schreurs M.W.J., Terlouw S., Gerth van Wijk R., Wichers H.J. (2023). Tolerance Induction in Cow’s Milk Allergic Children by Heated Cow’s Milk Protein: The iAGE Follow-Up Study. Nutrients.

[46] Inuo C., Tanaka K., Suzuki S., Nakajima Y., Yamawaki K., Tsuge I., Urisu A., Kondo Y. (2018). Oral Immunotherapy Using Partially Hydrolyzed Formula for Cow’s Milk Protein Allergy: A Randomized, Controlled Trial. Int. Arch. Allergy Immunol..

[47] Burks A.W., Jones S.M., Wood R.A., Fleischer D.M., Sicherer S.H., Lindblad R.W., Stablein D., Henning A.K., Vickery B.P., Liu A.H. (2012). Consortium of Food Allergy Research (CoFAR). Oral immunotherapy for treatment of egg allergy in children. N. Engl. J. Med..

[48] Kim E.H., Jones S.M., Burks A.W., Wood R.A., Sicherer S.H., Leung D.Y.M., Henning A.K., Lindblad R.W., Dawson P., Keet C. (2020). A 5-year summary of real-life dietary egg consumption after completion of a 4-year egg powder oral immunotherapy (eOIT) protocol. J. Allergy Clin. Immunol..

[49] Palosuo K., Karisola P., Savinko T., Fyhrquist N., Alenius H., Mäkelä M.J. (2021). A Randomized, Open-Label Trial of Hen’s Egg Oral Immunotherapy: Efficacy and Humoral Immune Responses in 50 Children. J. Allergy Clin. Immunol. Pract..

[50] Itoh-Nagato N., Inoue Y., Nagao M., Fujisawa T., Shimojo N., Iwata T., J-OIT Group (2018). Desensitization to a whole egg by rush oral immunotherapy improves the quality of life of guardians: A multicenter, randomized, parallel-group, delayed-start design study. Allergol. Int..

[51] Bloom K.A., Huang F.R., Bencharitiwong R., Bardina L., Ross A., Sampson H.A., Nowak-Węgrzyn A. (2014). Effect of heat treatment on milk and egg proteins allergenicity. Pediatr. Allergy Immunol..

[52] Dang T.D., Peters R.L., Allen K.J. (2016). Debates in allergy medicine: Baked egg and milk do not accelerate tolerance to egg and milk. World Allergy Organ. J..

[53] Leonard S.A. (2016). Debates in allergy medicine: Baked milk and egg ingestion accelerates resolution of milk and egg allergy. World Allergy Organ. J..

[54] Palosuo K., Kukkonen A.K., Pelkonen A.S., Mäkelä M.J. (2018). Gal d 1-specific IgE predicts allergy to heated egg in Finnish children. Pediatr. Allergy Immunol..

[55] Lazizi S., Labrosse R., Graham F. (2022). Transitioning peanut oral immunotherapy to clinical practice. Front. Allergy.

[56] Varshney P., Jones S.M., Scurlock A.M., Perry T.T., Kemper A., Steele P., Hiegel A., Kamilaris J., Carlisle S., Yue X. (2011). A randomized controlled study of peanut oral immunotherapy: Clinical desensitization and modulation of the allergic response. J. Allergy Clin. Immunol..

[57] Anagnostou K., Islam S., King Y., Foley L., Pasea L., Bond S., Palmer C., Deighton J., Ewan P., Clark A. (2014). Assessing the efficacy of oral immunotherapy for the desensitization of peanut allergy in children (STOP II): A phase 2 randomized controlled trial. Lancet.

[58] Vickery B.P., Berglund J.P., Burk C.M., Fine J.P., Kim E.H., Kim J.I., Keet C.A., Kulis M., Orgel K.G., Guo R. (2017). Early oral immunotherapy in peanut-allergic preschool children is safe and highly effective. J. Allergy Clin. Immunol..

[59] Jones S.M., Kim E.H., Nadeau K.C., Nowak-Wegrzyn A., Wood R.A., Sampson H.A., Scurlock A.M., Chinthrajah S., Wang J., Pesek R.D. (2022). Efficacy and safety of oral immunotherapy in children aged 1–3 years with peanut allergy (the Immune Tolerance Network IMPACT trial): A randomized placebo-controlled study. Lancet.

[60] Bird J.A., Spergel J.M., Jones S.M., Rachid R., Assa’ad A.H., Wang J., Leonard S.A., Laubach S.S., Kim E.H., Vickery B.P. (2018). ARC001 Study Group. Efficacy and Safety of AR101 in Oral Immunotherapy for Peanut Allergy: Results of ARC001, a Randomized, Double-Blind, Placebo-Controlled Phase 2 Clinical Trial. J. Allergy Clin. Immunol. Pract..

[61] Vickery B.P., Vereda A., Casale T.B., Beyer K., du Toit G., Hourihane J.O., Jones S.M., Shreffler W.G., Marcantonio A., Zawadzki R. (2018). PALISADE Group of Clinical Investigators. AR101 Oral Immunotherapy for Peanut Allergy. N. Engl. J. Med..

[62] Vickery B.P., Vereda A., Nilsson C., du Toit G., Shreffler W.G., Burks A.W., Jones S.M., Fernández-Rivas M., Blümchen K., Hourihane J.O. (2021). Continuous and Daily Oral Immunotherapy for Peanut Allergy: Results from a 2-Year Open-Label Follow-On Study. J. Allergy Clin. Immunol. Pract..

[63] Fernandez-Rivas M., Vereda A., Vickery B.P., Sharma V., Nilsson C., Muraro A., Hourihane J.O., DunnGalvin A., du Toit G., Blumchen K. (2022). Open-label follow-on study evaluating the efficacy, safety, and quality of life with extended daily oral immunotherapy in children with peanut allergy. Allergy.

[64] Nowak-Węgrzyn A., Wood R.A., Nadeau K.C., Pongracic J.A., Henning A.K., Lindblad R.W., Beyer K., Sampson H.A. (2019). Multicenter, randomized, double-blind, placebo-controlled clinical trial of vital wheat gluten oral immunotherapy. J. Allergy Clin. Immunol..

[65] Nguyen K., Lewis M.O., Hanna E., Alfaro M.K.C., Corrigan K., Buonanno J., Datta R., Brown-Whitehorn T., Spergel J.M., Cianferoni A. (2023). Safety of Multifood Oral Immunotherapy in Children Aged 1 to 18 Years at an Academic Pediatric Clinic. J. Allergy Clin. Immunol. Pract..

[66] Howe L.C., Leibowitz K.A., Perry M.A., Bitler J.M., Block W., Kaptchuk T.J., Nadeau K.C., Crum A.J. (2019). Changing Patient Mindsets about Non-Life-Threatening Symptoms During Oral Immunotherapy: A Randomized Clinical Trial. J. Allergy Clin. Immunol. Pract..

[67] Mempel M., Rakoski J., Ring J., Ollert M. (2003). Severe anaphylaxis to kiwi fruit: Immunologic changes related to successful sublingual allergen immunotherapy. J. Allergy Clin. Immunol..

[68] Kinaciyan T., Nagl B., Faustmann S., Frommlet F., Kopp S., Wolkersdorfer M., Wöhrl S., Bastl K., Huber H., Berger U. (2018). Efficacy and safety of 4 months of sublingual immunotherapy with recombinant Mal d 1 and Bet v 1 in patients with birch pollen-related apple allergy. J. Allergy Clin. Immunol..

[69] Enrique E., Pineda F., Malek T., Bartra J., Basagaña M., Tella R., Castelló J.V., Alonso R., de Mateo J.A., Cerdá-Trias T. (2005). Sublingual immunotherapy for hazelnut food allergy: A randomized, double-blind, placebo-controlled study with a standardized hazelnut extract. J. Allergy Clin. Immunol..

[70] Garrido-Fernández S., García B.E., Sanz M.L., Echechipía S., Lizaso M.T., Tabar A.I. (2014). Are basophil activation and sulphidoleukotriene determination useful tests for monitoring patients with peach allergy receiving sublingual immunotherapy with a Pru p 3-enriched peach extract?. J. Investig. Allergol. Clin. Immunol..

[71] Keet C.A., Frischmeyer-Guerrerio P.A., Thyagarajan A., Schroeder J.T., Hamilton R.G., Boden S., Steele P., Driggers S., Burks A.W., Wood R.A. (2012). The safety and efficacy of sublingual and oral immunotherapy for milk allergy. J. Allergy Clin. Immunol..

[72] Kim E.H., Bird J.A., Kulis M., Laubach S., Pons L., Shreffler W., Steele P., Kamilaris J., Vickery B., Burks A.W. (2011). Sublingual immunotherapy for peanut allergy: Clinical and immunologic evidence of desensitization. J. Allergy Clin. Immunol..

[73] Kim E.H., Yang L., Ye P., Guo R., Li Q., Kulis M.D., Burks A.W. (2019). Long-term sublingual immunotherapy for peanut allergy in children: Clinical and immunologic evidence of desensitization. J. Allergy Clin. Immunol..

[74] Kim E.H., Keet C.A., Virkud Y.V., Chin S., Ye P., Penumarti A., Smeekens J., Guo R., Yue X., Li Q. (2023). Open-label study of the efficacy, safety, and durability of peanut sublingual immunotherapy in peanut-allergic children. J. Allergy Clin. Immunol..

[75] Fleischer D.M., Burks A.W., Vickery B.P., Scurlock A.M., Wood R.A., Jones S.M., Sicherer S.H., Liu A.H., Stablein D., Consortium of Food Allergy Research (CoFAR) (2013). Sublingual immunotherapy for peanut allergy: A randomized, double-blind, placebo-controlled multicenter trial. J. Allergy Clin. Immunol..

[76] Burks A.W., Wood R.A., Jones S.M., Sicherer S.H., Fleischer D.M., Scurlock A.M., Vickery B.P., Liu A.H., Henning A.K., Consortium of Food Allergy Research (2015). Sublingual immunotherapy for peanut allergy: Long-term follow-up of a randomized multicenter trial. J. Allergy Clin. Immunol..

[77] Narisety S.D., Frischmeyer-Guerrerio P.A., Keet C.A., Gorelik M., Schroeder J., Hamilton R.G., Wood R.A. (2015). A randomized, double-blind, placebo-controlled pilot study of sublingual versus oral immunotherapy for the treatment of peanut allergy. J. Allergy Clin. Immunol..

[78] Taylor S.L., Moneret-Vautrin D.A., Crevel R.W., Sheffield D., Morisset M., Dumont P., Remington B.C., Baumert J.L. (2010). Threshold dose for peanut: Risk characterization based upon diagnostic oral challenge of a series of 286 peanut-allergic individuals. Food Chem. Toxicol..

[79] Baumert J.L., Taylor S.L., Koppelman S.J. (2018). Quantitative Assessment of the Safety Benefits Associated with Increasing Clinical Peanut Thresholds Through Immunotherapy. J. Allergy Clin. Immunol. Pract..

[80] Jones S.M., Burks A.W., Dupont C. (2014). State of the art on food allergen immunotherapy: Oral, sublingual, and epicutaneous. J. Allergy Clin. Immunol..

[81] Sampson H.A., Shreffler W.G., Yang W.H., Sussman G.L., Brown-Whitehorn T.F., Nadeau K.C., Cheema A.S., Leonard S.A., Pongracic J.A., Sauvage-Delebarre C. (2017). Effect of Varying Doses of Epicutaneous Immunotherapy vs Placebo on Reaction to Peanut Protein Exposure Among Patients with Peanut Sensitivity: A Randomized Clinical Trial. JAMA.

[82] Parrish C.P. (2018). Management of peanut allergy: A focus on novel immunotherapies. Am. J. Manag. Care.

[83] Gupta R.S., Warren C.M., Smith B.M., Blumenstock J.A., Jiang J., Davis M.M., Nadeau K.C. (2018). The Public Health Impact of Parent-Reported Childhood Food Allergies in the United States. Pediatrics.

[84] Greenhawt M., Marsh R., Gilbert H., Sicherer S., DunnGalvin A., Matlock D. (2018). Understanding caregiver goals, benefits, and acceptable risks of peanut allergy therapies. Ann. Allergy Asthma Immunol..

[85] Kansen H.M., Le T.M., Knulst A.C., Gorissen D.M.W., van der Ent C.K., Meijer Y., van Erp F.C. (2020). Three-year follow-up after peanut food challenges: Accidental reactions in allergic children and introduction failure in tolerant children. J. Allergy Clin. Immunol..

[86] Jones S.M., Sicherer S.H., Burks A.W., Leung D.Y., Lindblad R.W., Dawson P., Henning A.K., Berin M.C., Chiang D., Consortium of Food Allergy Research (2017). Epicutaneous immunotherapy for the treatment of peanut allergy in children and young adults. J. Allergy Clin. Immunol..

[87] Scurlock A.M., Burks A.W., Sicherer S.H., Leung D.Y.M., Kim E.H., Henning A.K., Dawson P., Lindblad R.W., Berin M.C., Consortium for Food Allergy Research (CoFAR) (2021). Epicutaneous immunotherapy for treatment of peanut allergy: Follow-up from the Consortium for Food Allergy Research. J. Allergy Clin. Immunol..

[88] Fleischer D.M., Greenhawt M., Sussman G., Bégin P., Nowak-Wegrzyn A., Petroni D., Beyer K., Brown-Whitehorn T., Hebert J., Hourihane J.O. (2019). Effect of Epicutaneous Immunotherapy vs. Placebo on Reaction to Peanut Protein Ingestion Among Children with Peanut Allergy: The PEPITES Randomized Clinical Trial. JAMA.

[89] Fleischer D.M., Shreffler W.G., Campbell D.E., Green T.D., Anvari S., Assa’ad A., Bégin P., Beyer K., Bird J.A., Brown-Whitehorn T. (2020). Long-term, open-label extension study of the efficacy and safety of epicutaneous immunotherapy for peanut allergy in children: PEOPLE 3-year results. J. Allergy Clin. Immunol..

[90] Greenhawt M., Sindher S.B., Wang J., O’Sullivan M., du Toit G., Kim E.H., Al-bright D., Anvari S., Arends N., Arkwright P.D. (2023). Phase 3 Trial of Epicutane-ous Immunotherapy in Toddlers with Peanut Allergy. N. Engl. J. Med..

[91] Dupont C., Kalach N., Soulaines P., Legoué-Morillon S., Piloquet H., Benhamou P.H. (2010). Cow’s milk epicutaneous immunotherapy in children: A pilot trial of safety, acceptability, and impact on allergic reactivity. J. Allergy Clin. Immunol..

[92] Spergel J.M., Elci O.U., Muir A.B., Liacouras C.A., Wilkins B.J., Burke D., Lewis M.O., Brown-Whitehorn T., Cianferoni A. (2020). Efficacy of Epicutaneous Immunotherapy in Children with Milk-Induced Eosinophilic Esophagitis. Clin. Gastroenterol. Hepatol..

[93] Pongracic J.A., Gagnon R., Sussman G., Siri D., Oriel R.C., Brown-Whitehorn T.F., Green T.D., Campbell D.E., Anvari S., Berger W.E. (2022). Safety of Epicutaneous Immunotherapy in Peanut-Allergic Children: REALISE Randomized Clinical Trial Results. J. Allergy Clin. Immunol. Pract..

[94] Davis C.M., Lange L., Beyer K., Fleischer D.M., Ford L., Sussman G., Oriel R.C., Pongracic J.A., Shreffler W., Bee K.J. (2022). Efficacy and safety of peanut epicuta-neous immunotherapy in patients with atopic comorbidities. J. Allergy Clin. Immunol. Glob..

[95] Brandström J., Vetander M., Sundqvist A.C., Lilja G., Johansson S.G.O., Melén E., Sverremark-Ekström E., Nopp A., Nilsson C. (2019). Individually dosed omalizumab facilitates peanut oral immunotherapy in peanut allergic adolescents. Clin. Exp. Allergy.

[96] Leung D.Y., Sampson H.A., Yunginger J.W., Burks A.W., Schneider L.C., Wortel C.H., Davis F.M., Hyun J.D., Shanahan W.R., Avon Longitudinal Study of Parents and Children Study Team (2003). Effect of anti-IgE therapy in patients with peanut allergy. N. Engl. J. Med..

[97] MacGinnitie A.J., Rachid R., Gragg H., Little S.V., Lakin P., Cianferoni A., Heimall J., Makhija M., Robison R., Chinthrajah R.S. (2017). Omalizumab facilitates rapid oral desensitization for peanut allergy. J. Allergy Clin. Immunol..

[98] Sindher S.B., Kumar D., Cao S., Purington N., Long A., Sampath V., Zedeck S.S., Woch M.A., Garcia-Lloret M., Chinthrajah R.S. (2022). Phase 2, randomized multi oral immunotherapy with omalizumab ‘real life’ study. Allergy.

[99] Zuberbier T., Wood R.A., Bindslev-Jensen C., Fiocchi A., Chinthrajah R.S., Worm M., Deschildre A., Fernandez-Rivas M., Santos A.F., Jaumont X. (2023). Omalizumab in IgE-Mediated Food Allergy: A Systematic Review and Meta-Analysis. J. Allergy Clin. Immunol. Pract..

[100] Sindher S.B., Hillier C., Anderson B., Long A., Chinthrajah R.S. (2023). Treatment of food allergy: Oral immunotherapy, biologics, and beyond. Ann. Allergy Asthma Immunol..

[101] Ibáñez-Sandín M.D., Escudero C., Candón Morillo R., Lasa E.M., Marchán-Martín E., Sánchez-García S., Terrados S., González Díaz C., Juste S., Martorell A. (2021). Oral immunotherapy in severe cow’s milk allergic patients treated with omalizumab: Real life survey from a Spanish registry. Pediatr. Allergy Immunol..

[102] Worm M., Francuzik W., Dölle-Bierke S., Alexiou A. (2021). Use of biologics in food allergy management. Allergol. Sel..

[103] de Silva D., del Río Rodríguez P., de Jong N.W., Khaleva E., Singh C., Nowak-Wegrzyn A., Muraro A., Begin P., Pajno G., Fiocchi A. (2022). Allergen immunotherapy and/or biologicals for IgE-mediated food allergy: A systematic review and meta-analysis. Allergy.

[104] Sindher S.B., Barshow S., Tirumalasetty J., Arasi S., Atkins D., Bauer M., Bégin P., Collins M.H., Deschildre A., Doyle A.D. (2023). The role of biologics in pediatric food allergy and eosinophilic gastrointestinal disorders. J. Allergy Clin. Immunol..

[105] Yanagida N., Sato S., Asaumi T., Okada Y., Ogura K., Ebisawa M. (2015). A Single-Center, Case-Control Study of Low-Dose-Induction Oral Immunotherapy with Cow’s Milk. Int. Arch. Allergy Immunol..

[106] Reisacher W.R., Suurna M.V., Rochlin K., Bremberg M.G., Tropper G. (2016). Oral mucosal immunotherapy for allergic rhinitis: A pilot study. Allergy Rhinol..

